# Yellow Fever: Precautions to Be Taken in Countries Where It Is Prevalent

**Published:** 1901-12-28

**Authors:** Louis Vintras

**Affiliations:** Physician to the French Hospital


### Hospital Clinics and Medical Progress.

### YELLOW FEVER: PRECAUTIONS TO BE TAKEN IN COUNTRIES WHERE IT IS PREVALENT.

By Louis Vintras, M.D., B.Sc., Physician to the French Hospital.

Along all the western coast and part of the eastern coast of Central and South America there are now numerous English hospitals, the medical and nursing staffs of which are English and drawn from here. Medical men and nurses are thus continually going to these parts where yellow fever, especially in Brazil, is more or less endemic. The same takes place as regards the large colonial hospitals of the West Indies, though in justice to these colonies it should be said that of late years they have mostly been free from the scourge, and that when it has made its appearance the epidemics have been mild and of short duration. Having been a continuous visitor to these parts of late years, the above facts suggested to me that it might be of interest to the readers of The Hospital, to know something of the precautions necessary to be observed by those called upon to reside there. It is well known that new-comers are more prone to catch the disease than those who have resided in hot climates for some time, but among white people those with fair hair and fair skins are most liable. Again, strange as it may seem, it is the plethoric constitutions, people of a florid, active habit of life, who, when attacked, succumb most readily, while the comparatively less robust escape. The active, healthy, full-blooded northerner going to these countries, where the climate is enervating and where the great heat of the day precludes almost entirely the possibility of outdoor exercise and, indeed, leaves little inclination for it, falls, unfortunately, too often a victim to yellow fever. With due care, however, and attention to diet and clothing much of the danger is removed, and it must be admitted that many of the sad cases on record are largely due to indifference to the details of life and too much reliance on constitutional strength. Many Europeans and English people especially, taking up their abode in the tropics, live in the same way as they would at home, keeping to a heavy meat diet, and drinking beer or wine. It stands to reason that in a hot climate, where the vitality is lowered by excessive perspiration, where healthy exercise is neglected or impossible, the continuous digestion of heavy food throws a strain on the digestive organs and in time weakens them. Gastric trouble, an irritable condition of the intestinal tract, soon appears; the liver becomes sluggish, congested, with the natural sequelæ of heaviness, disinclination for work and mental depression. In the case of a man, he too often seeks by increasing his daily quantum of alcohol to counteract these symptoms, with the result that he only adds another factor to those already working against him. But in all cases where the intestinal tract is weakened, the liver working unsatisfactorily, the secretion of bile diminishing and constipation becoming chronic, the system presents an almost perfect nidus for the reception and elaboration of yellow fever. The appearance of these symptoms should always be looked upon with alarm and no time lost in combating them. A full dose of castor oil—the best and safest of all purgatives in these cases—should be taken at once; for 48 hours or more the diet should consist of milk and beef tea, taken frequently, with an ounce of brandy or whisky twice or three times a day. Wine, beer, aërated waters should be strictly avoided and the patient should, if possible, keep indoors, or at least not venture out after sunset. It is no good waiting, putting it off until the morrow; for it is astonishing how rapidly such a condition, which at home would hardly be given a thought, will in the tropics pass into one of the extremest danger and possibly prove fatal. It is not uncommon in Brazil, in those parts where yellow fever is always lurking, or anywhere where the disease has made its appearance, to meet a European one day who complains of having felt out of sorts of late and the next morning to hear that he is dead. Next, and of great importance also, is the question of dress. One of the charms of the tropics is that there one dresses to suit the climate. Linen or duck clothes, or the thinnest alpacas are worn by both sexes during the day, and it is this which makes visitors out there say that they suffer less from the heat than they do on a hot summer's day in England. There is no harm in clothes of the lightest description, *so long as they are only worn during the heat of the day* and changed immediately for a suit or dress of cloth or some warmer material as soon as the sun goes down. But such is the comfort of these light vestments that one is tempted to retain them after the heat of the day, to enjoy the more the welcome cool of the evening. This is dangerous in the extreme; for with the departure of the sun a clammy mist rises from the earth, making one's clothes limp and moist, and in the case of linen or duck finding its way direct to the skin, producing a chill. In those climates a chill does not mean a cold in the head or a slight bronchial catarrh, it means *fever*—probably malarial, possibly even more serious. In any case the sudden and often excessive loss of animal heat, which coincides with the equally sudden termination of the day, is not one of the least among the strains which the constitution has to bear in the tropics. The mere fact that after dark the thermometer may still register what we should at home look upon as an excessive temperature does not alter the effect on the system of the sudden change and dampness. Sudden changes of temperature are always trying; we only take note of them in the lower portion of the register, but I believe that a drop from 90° to 80° is as trying to the constitution as a drop from 50° to 40°. It is after sunset that the body requires nourishment and that the heavy meal of the day should be taken, mostly consisting of hot dishes. Ice, which may be taken in considerable quantities during the day time, should now be used with care and a little alcohol mixed with the iced drink. Fruit, especially melons, which are very plentiful throughout the tropics, should be avoided; indeed, most South Americans never touch fruit after dark, and I believe the same to be a golden rule in the corresponding latitudes of Africa. The evening mist, which is so universally prevalent in these parts, is not only dangerous from its chilliness but also, perhaps, still more for another reason. It is almost proved beyond dispute that the germs of both malaria and yellow fever are fostered in the damp soil of the tropics, and that the rising mist may bear them up with it, and people should be most careful in those climates never to sit or lie on the ground after dark. It is also a common belief that newcomers should not unnecessarily expose themselves to the full blaze of the sun, for though in the West Indies and in the hottest parts of South America sunstroke is rare, the intense heat is said to have a tendency to produce changes in the blood, thinning its consistency. The absolute truth of this statement I have never been able to verify with any exactitude; as the want of consistency of the blood often met with among white people out there may be due to other causes; but the idea is widespread and worth consideration, as it may have an influence on hepatic elaboration, and I think it must be conceded as certain that it is the liver which is primarily affected by the poison of yellow fever—indeed, the local lesion of this fever is a functional hepatic perturbation. That these climates are abnormally dangerous to the average European is not by any means the impression I wish to convey, nor that yellow fever is in itself necessarily fatal. The West Indies present indeed in some respects an almost ideal climate; hundreds of Englishmen and Englishwomen make their homes in the great towns along the coast of South America, and the number of cases of yellow fever among them is comparatively small. But it is certainly a danger ever possible in those parts, a danger to be borne in mind, but not exaggerated. Those who go there should not live in undue dread of the disease appearing, nor when it is present give way to panic. Fear and panic react through the nervous system, on the constitution generally, and lower its stamina, thus aiding and abetting in the spread of the scourge. I have said enough, I think, to give those called upon to reside in those climates an idea of the primary precautions to be adopted against the possibility of infection by yellow fever, and to show at the same time that in case of infection, if the first symptoms be promptly attended to, there need be no reason to fear a fatal issue.

